# Management of hypothyroidism with combination thyroxine (T4) and triiodothyronine (T3) hormone replacement in clinical practice: a review of suggested guidance

**DOI:** 10.1186/s13044-018-0045-x

**Published:** 2018-01-17

**Authors:** Colin Dayan, Vijay Panicker

**Affiliations:** 1 Thyroid Research Group, School of Medicine, Cardiff University, Cardiff, UK; 2 Department of Endocrinology, Sir Charles Gairdner Hospital, Nedlands, WA 6009 Australia

**Keywords:** Levothyroxine, Liothyronine, T4, T3, Thyroid hormone replacement

## Abstract

**Background:**

Whilst trials of combination levothyroxine/liothyronine therapy versus levothyroxine monotherapy for thyroid hormone replacement have not shown any superiority, there remains a small subset of patients who do not feel well on monotherapy. Whilst current guidelines do not suggest routine use of combination therapy they do acknowledge a trial in such patients may be appropriate. It appears that use of combination therapy and dessicated thyroid extract is not uncommon but often being used by non-specialists and not adequately monitored. This review aims to provide practical advice on selecting patients, determining dose and monitoring of such a trial.

**Main body:**

It is important to select the correct patient for a trial so as to not delay diagnosis or potentially worsen an undiagnosed condition. An appropriate starting dose may be calculated but accuracy is limited by available formulations and cost. Monitoring of thyroid function, benefits and adverse effects are vital in the trial setting given lack of evidence of safe long term use. Also important is that patients understand set up of the trial, potential risks involved and give consent.

**Conclusion:**

Whilst evidence is lacking on whether a small group of patients may benefit from combination therapy a trial may be indicated in those who remain symptomatic despite adequate levothyroxine monotherapy. This should be undertaken by clinicians experienced in the field with appropriate monitoring for adverse outcomes in both short and long term.

## Background

Since the 1970s levothyroxine (LT4) has become the standard of care for thyroid hormone replacement in subjects unable to produce their own thyroid hormones due to congenital, autoimmune or iatrogenic causes. Levothyroxine has become one of the most widely used drugs worldwide, it is the most commonly prescribed drug in the United States (US), the third most in the United Kingdom (UK), and there is evidence that its use is steadily increasing [1, 2]. Despite this, there remains controversy as to the best way to therapeutically replace thyroid hormones, with a small group of people on LT4 monotherapy not feeling as though they have achieved their premorbid well-being [3, 4] and hence a significant push for other therapies including combination LT4/liothyronine (LT3) therapy, LT3 alone or extracts from animal thyroids.

Possible reasons for some patients not responding to levothyroxine monotherapy and evidence for efficacy of mono- and combination therapy have been well covered in recent systematic reviews by both the American Thyroid Association (ATA) [5] and European Thyroid Association (ETA) [6]. Both guidelines suggest that in select cases, a trial of therapy including LT3 can be considered, carefully supervised by an expert in the field. The purpose of this review, therefore, is not to repeat the analysis in these recent reports, but to provide a more practical approach to the use of combination therapy in clinical practice including safety aspects and cost, where a trial is considered appropriate.

The target audience for this review is clinicians who incorporate or would consider incorporating trials of combination LT4/LT3 therapy in their practice, or who have patients who have questions about this therapy. The review was performed by literature search in each area, review of published articles and studies and practical experience.

## Thyroid hormone replacement

Although the thyroid gland produces T4 and T3, LT4 monotherapy has been the mainstay of thyroid hormone replacement since the 1970s replacing desiccated thyroid extract (DTE) which had been used for many years prior. This is because it is easily administered, well absorbed by the oral route and its long half-life allows for once daily dosing with very stable serum levels. It was also shown to be converted to T3 within the body [7] alleviating the need to add LT3 which does not have the same stable pharmacological profile. Furthermore, it has also been shown that the majority of circulating T3 comes from peripheral conversion of T4 to T3 and not secretion of T3 from the thyroid [8], hence a T4:T3 secretion ratio of approximately 14:1 appears average in humans, suggesting only a small role for secreted T3. However, there is a small group of patients who do not feel back to their euthyroid well-being despite having thyroid function tests suggestive of adequate replacement on LT4 [3, 9, 10]. There are also several studies showing that on LT4 monotherapy serum T3 levels are significantly lower for the same TSH in euthyroid patients [11–16], although the clinical significance of this is unknown. Another study showed it was not possible to normalise serum TSH, T3 and T4 levels or tissue T3 levels in laboratory animals giving them LT4 monotherapy [17].

Because of this there have now been at least 13 randomized controlled trials (RCT) comparing efficacy of combination LT4/LT3 therapy versus LT4 monotherapy for thyroid hormone replacement [18–30]. There has also been one trial comparing DTE to LT4 monotherapy [31]. The trials differ significantly in study population size, method of substituting LT3 for LT4, dose, length of study and outcomes measured. There have now been 4 systematic reviews/meta-analyses of these studies attempting to clarify the findings [32–35]. Overall these meta-analyses have suggested there is no significant benefit of combination LT4/LT3 therapy compared to LT4 monotherapy in terms of mood, health-related quality of life or cognitive function. This provides reasonable evidence that at a population level there is no benefit of using combination therapy over monotherapy. However, the different methods of replacing LT4 with LT3 (some resulting in raised TSH suggestive of under replacement) and small size and hence power of some studies should be considered. There was no increase in adverse events in the combination groups in this admittedly short period of follow up. There is one randomized cross-over trial comparing DTE with levothyroxine monotherapy in 70 patients for 16 weeks each arm [31]. This did not show any significant benefit of DTE over LT4 in symptoms and neurocognitive markers. There was an increase in preference for DTE over LT4 however it is unclear what this signifies given an overall lack of improvement in symptoms. The study was too small to pick up subgroups responding to T3 and future studies in this area may consider having a synthetic T4/T3 arm also to determine if any benefit is due to the addition of T3 or DTE itself.

Whilst this evidence is convincing there remains a possibility that a small subset of hypothyroid patients will do better on combination therapy; this group may get lost in the larger group of patients with no benefit. Practicing clinicians will be able to identify a group of patients not satisfied on LT4 monotherapy which makes up a small subset of all their patients on LT4. There is also one study suggesting that a polymorphism in the DIO2 gene which codes for the deiodinase 2 enzyme (Thr92Ala), important for conversion of T4 to T3 in many tissues including the brain may suggest the group which will respond to therapy (rare homozygotes approximately 12% of the Caucasian population) [36]. These findings remain controversial however, particularly with inconsistent findings of the functional effect of this polymorphism [37–39].

After extensive review of available literature on this topic guidelines were produced by both the ATA and ETA related to thyroid hormone replacement with LT4 and alternatives. Both guidelines suggest there is insufficient or no strong evidence of superiority of combination LT4/LT3 therapy over LT4 monotherapy in patients with hypothyroidism. However, both acknowledge that there are patients who have persistent symptoms or sub-optimal health despite LT4 therapy guided by normal thyroid biochemistry and suggest possible reasons for this include inadequacy of LT4 monotherapy to normalise serum and tissue T4 and T3 levels. Thus, both suggest that in an appropriate clinical setting (see below) combination therapy may be trialled to determine if it is beneficial for the individual patient [5, 6]. The remainder of this review will consider practical issues related to such a trial/long term therapy.

As in all clinical practice, clinicians should only offer treatments that they are comfortable using. The experience is, however, that patients wishing for a trial are often frustrated at the lack of specialists willing to provide such, and therefore often obtain combination therapy from health practitioners with little training in the area, who do not monitor for complications, can give incorrect doses/dose ratios and offer ongoing treatment without assessing the benefits. In some instances, patients self-treat with medications obtained online. Until more is known of benefits and risks, LT3 should not be offered routinely. LT3 should only be trialled in pts. who specifically request it, who have marked persisting symptoms, and who fully understand and accept (with written/documented information if possible) the unknown potential for long-term harm. Endocrinologists are clearly best suited to provide this.

## Current practice

One of the reasons for this review was that despite recommendations and guidelines from various specialist bodies, use of combination T4/T3 therapy appears significant in most developed countries. In a survey of specialist members of The Endocrine Society, The American Thyroid Association and The American Association of Clinical Endocrinologists spread internationally, but mostly in North America; showed that 3.6% of the 880 respondents would trial adding LT3 to LT4 in the setting of a hypothyroid patient with persistent symptoms despite a TSH within the target range [40]. De Jong and colleagues from the Netherlands using data from a sample of Dutch pharmacies showed that use of combination T4/T3 was 0.82% of thyroid hormone users in 2005 and this slowly rose to 0.90% by 2011 [41]. In the TEARS study population sampled from an area in Scotland 0.95% of those on thyroid hormone replacement were taking combination T4/T3 therapy and 0.21% T3 only [42]. A study by Michaelsson and colleagues from Denmark which elicited survey responses from patients known to be on T4/T3 combinations did not estimate the frequency of combination use, but did show that in the responding population of 293 on combination therapy slightly more patients had received their treatment from their GP (42%) than had from their endocrinologist (39%), 50% were on DTE with 43% on T4/T3 and 28% were adjusting their own dose [43]. This study suffers from selection bias associated with internet surveys particularly on a topic which has been strongly debated publicly recently in Denmark, however, it does highlight that methods other than LT4 monotherapy for thyroid hormone replacement may frequently not be monitored by specialists.

*Practice point: Alternatives to LT4 monotherapy for thyroid hormone replacement are being used in a small percentage of hypothyroid patients and it appears frequently without specialist oversight and appropriate monitoring. If specialists are willing to discuss the available evidence, possible benefits and adverse effects of such therapy with patients, it is likely to make this practice safer.*

## Patient selection

Patient selection is important to determine pre-trial whether the patient is likely to gain benefit from the treatment and prevent harm to the patient. There are circumstances in which a trial may delay another treatable diagnosis or put the patient at significant risk without possibility of benefit in which it may be inappropriate.

### Collect evidence that there is thyroid dysfunction

The first important step in this age of accessibility to thyroid function testing is to clarify the diagnosis of hypothyroidism requiring thyroid hormone replacement. There is good evidence from the UK that the median TSH level at which LT4 therapy was commenced is relatively low (Taylor et al. 7.8 mU/L in 2009 [2], Leese et al. 6.2 mU/L in 2001 [44]). It is therefore important to clarify the initial diagnosis, if possible with access to diagnosis thyroid function/antibody levels and presenting symptoms. It is also worthwhile considering if there was an initial response to LT4 therapy which was subsequently lost or if there was never a response.

### Ensure adequate dose of LT4 has been used

It is important to ensure that the patient is on adequate T4 dosage prior to a trial of combination therapy. Most guidelines would suggest returning the serum TSH level to the population reference range as an indication of adequate replacement [45, 46], as clinical symptoms have not been shown to be accurately predict serum levels [47]. However, even with these guidelines studies have shown that a high percentage of patients on T4 have serum TSH above the reference range, even those on for many years [2, 3, 47, 48]. Furthermore, many would suggest a serum TSH in the lower part of the reference range is appropriate, given the skewed distribution of TSH values in the reference population [49], and the concept that individual TSH levels within the reference range vary little in health and therefore each person may have a genetically derived set point [50]. There are however no trials suggesting this improves any measurable clinical markers compared with TSH within the entire reference range and this practice is not accepted by all [51]. Furthemore a trial looking at slight alterations in LT4 dose in hypothyroid patients did not show any difference between clinical symptoms or measurable parameters despite clear differences in TSH on the different treatment regimens [52].

### Exclude co-morbidities

Given the generalised nature of hypothyroid symptoms it is possible another condition may be causing them, or that the two conditions may co-exist, given the high prevalence of hypothyroidism in the population. Typical conditions which may lead to a mis-diagnosis are Depression, Chronic Fatigue Syndrome and Fibromyalgia. As the majority of hypothyroidism has an autoimmune origin and these conditions frequently occur with other autoimmune conditions [53–55] it is worthwhile considering whether there may be another diagnosis present with careful history taking, examination and targeted investigations if appropriate. The question of whether thyroid autoimmunity itself can cause symptoms is a more complicated one. A large population study from Norway suggests not [56], however, two smaller studies suggest an association unrelated to thyroid hormone levels [57, 58] giving it some plausibility. The answer is therefore not clear. Again, if persisting symptoms are related to thyroid autoimmunity then these would not be expected to resolve with combination therapy but should improve as antibody levels subside. However, due to the tenuous link it would appear inadvisable to be measuring thyroid antibody levels and using this as a deciding factor to initiate a trial of combination therapy.

### Be aware of psychological comorbidities

This is important because psychological comorbidities may confuse the diagnosis (depressive symptoms versus hypothyroid symptoms), affect the response to treatment and in some cases be worsened by the addition of LT3 (for example anxiety).

### Exclusions

Although there is little clear evidence most practitioners would exclude patients with significant cardiovascular disease or arrhythmia for a trial, given the potential to cause life-threatening side effects. Furthermore, given the importance of tight control of thyroid hormone levels, particularly T4, for the normal development of the foetus and progression of pregnancy, and lack of data showing LT3 can be safely used in pregnancy, it is not recommended in pregnant women or woman actively trying to conceive. Patients with poorly controlled anxiety and thyroid cancer requiring suppression of serum TSH may also fall into a worrying area. It is unclear whether combination therapy in disseminated thyroid cancer can adequately suppress TSH across a 24 h period, and therefore the individual patients’ prognosis and need for TSH suppression needs to be considered and discussed in this setting.

### Not currently useful

Is *genetic testing* a useful aid to patient selection? On current evidence there is no clear genetic test which will determine patients who will respond to therapy. Use of an unvalidated test may preclude some patients receiving a trial who may benefit and therefore it is not recommended. Further studies are required to delineate the actual genetic markers which may be useful for patient selection. In a similar way trials have not suggested a biochemical marker (including thyroid hormone levels) which will predict who will respond (Table 1).

**Table 1:** Pre-trial assessment of potential patients

1. Collect evidence of thyroid dysfunction	
2. Ensure adequate dosage of levothyroxine has been trialled	
3. Exclude co-morbidiies	
4. Be aware of psychological co-morbidities	
5. Exclusions	
Not currently useful	Genetics
	Biochemistry
	Symptom profile

## Pharmacology and formulations

Studies using both older [59–61] and newer [62] thyroid hormone assays have suggested a diurnal rhythm of free T3 and TSH in healthy subjects with no thyroid disease. These studies suggest a peak of T3 at around 4 am with a nadir between 3 to 5 pm; this appears to lag behind TSH levels by about 90 mins [62]. Given its long half-life it is not surprising that in most studies free T4 levels remain very stable throughout the day. Despite a statistically measurable diurnal variation in T3 the actual difference in T3 levels is low (11.2%) and the levels are effectively stable over a 24 h period. It would appear reasonable to mimic these levels if trying to appropriately replace thyroid hormones, particularly as there are few biomarkers which reliably suggest complete thyroid hormone replacement. However, currently this is limited by formulations of T3 which are available (Table 2 for a non-exhaustive list). In many countries, the 20μg table of liothyronine is the only available making accurate dosing very difficult. This is reflected in the combination T4/T3 trials which were not uniform in their method of replacing LT4 with T3, either using a 10:1 or 5:1 LT4:LT3 ratio, or replacing an amount of LT4 with from a fifth to a whole dose of LT3.

**Table 2:** Available formulations of LT3 and combination LT3/LT4

Name	T3 dose	T4 dose	Available
Cytomel	5, 25, 50mcg		US, Canada, Netherlands
Thybon	20, 100mcg		UK
Tertroxin	20 mcg		Australia, South Africa
Liotyr	5 mcg (soft gel)		Italy
Prothyroid	10 mcg	100 mcg	Germany
Novothyral	5/15/20 mcg	25/75/100 mcg	Several Europe
Thyreotom forte	10/30 mcg	40/120 mcg	Czech republic

**Fig. 1:**
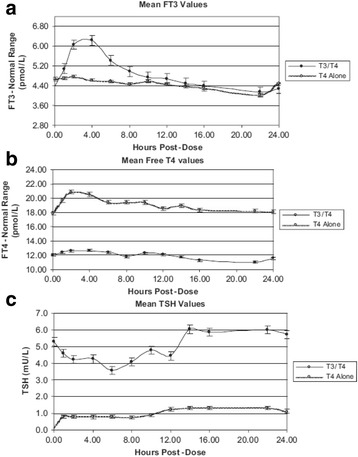
Mean values for **a** free T3, **b** free T4 and **c** TSH over a 24 h period in 10 patients with hypothyroidism on either LT4/LT3 combination or LT4 alone. From Saravanan et al. [63], used with permission

Studies looking at the pharmacology of LT3 replacement all show a significant peak of serum T3 2–4 h after dose and wearing off after 12 h in those on a single daily dose, these include hypothyroid patients on combination therapy [63] (Fig. 1), LT3 monotherapy [64, 65], and even euthyroid subjects taking LT3 only [66]. The profiles are very different to those in patient with normal endogenous thyroid function, and depending on the dose of LT3 the peak level is often above the reference range, and/or the serum TSH is raised/suppressed compared to T4 treatment [67]. Figure 1 suggests that to sample the peak serum T3 level a test taken 2–4 h after ingestion of the T3 would be appropriate. There is a study showing equivalent TSH responsiveness to TRH in patients on either T3 or T4 monotherapy [68], suggesting the rise in TSH seen in some of these studies is an indication of under replacement. Almost all the combination trials used once daily LT3 dosing with one using twice daily.

To truly mimic the normal production of T3 patients would have to split the dose of T3 and take it two or three times a day, however, the large dose size of the available products may preclude this in patients with a lower requirement for thyroid hormone. Comparison of the figures from Saravanan et al. [63] and Russell et al. [62] would suggest that to mimic serum T3 levels in euthyroid individuals the LT3 dose should be split with the second dose given approximately 8 h after the first. This may prevent the insomnia reported by some patients when they take LT3 prior to bed which is presumably secondary to a serum T3 peak whilst trying to sleep. What effect this will have on the steady state of the drug is unclear as there are no studies looking at serum levels in multiple daily dosing, and even the half-life of T3 is debated with a wide range of opinions. Saravanan et al. did not find any difference in cardiovascular parameters (pulse rate and blood pressure) between their groups on T4/T3 and T4 only despite the clear peak in T3 levels at approximately 4 h. This interesting finding reminds us that serum hormone levels do not necessarily reflect action in tissues due to the presence of thyroid hormone transporters and deiodinases in different tissues which may influence the effect of these hormones in individual tissues [69]. Furthermore, most actions of thyroid hormone take several hours to have effect as they require the synthesis of new mRNA and protein.

There are no commercially available slow release T3 formulations currently. Hennemann and colleagues tested their own slow release T3 preparation in combination with T4 and found that compared to once daily T3 there was a lower peak of T3 and smoother profile [70]. They suggested all future trials use slow release T3 but a commercially available product has not been forthcoming. Doubts have been raised as to whether this study showed a product that could be used as a once a day dosage and what actual effect it had given no change in TSH levels for either formulation [71]. Compounding pharmacies will provide compounded formulations of slow release T3, however the ATA do not recommend the use of compounded preparations except in cases of clear allergic reactions to commercial preparations [5]. This is because there are few studies on these products that meet scientific criteria for rigorous peer review showing that they can be used to provide adequate thyroid hormone replacement, are equivalent to approved preparations or have a long-term safety profile. Furthermore, the preparations need to be used relatively quickly after synthesis to prevent loss of efficacy due to degradation [72], requiring regular compounding of new products. In addition, compounded preparations are generally higher in cost and not standardised between different pharmacies. There have also been reports of thyrotoxicosis [73, 74] and hypothyroidism [75] caused by errors in compounding.

*Practice point: be aware of formulations of LT3 and LT4/LT3 available locally and if possible their pharmacokinetics. Errors in the preparations of compounded preparations have been reported.*

## Dosage

Wiersinga et al. in their review suggest 3 different methods for calculating appropriate dosages for combination T4/T3 therapy [6]. These are based on the assumptions that persisting symptoms are due to LT4 monotherapy being unable to deliver normal serum and tissue T4 and T3 levels in humans as shown in rats [17] and that mimicking the normal thyroid secretion of T4 and T3 will correct this [76]. The methods are based on using the dose of LT4 which gives the target TSH in the patient, then replacing a small amount of T4 with T3 using a 3:1 equivalence ratio derived from a study in thyroidectomized patients [77] to give the appropriate ratio. The three methods give a final dose T4:T3 ratio between 13:1 and 20:1, much closer to normal human thyroid secretion [8] but in generally lower than those used in the T4/T3 studies, in some cases significantly lower. Furthermore, many of the studies had variable ratios due to a fixed substitution (eg. 10μg T3 for 50μg T4). This dose ratio is also significantly lower than that of animal thyroid extracts in which the T4:T3 ratio is generally around 4:1 (see later). Note also however that for a patient previously on 100μg of T4 a day the T3 dose from these methods is between 4 and 6μg a day which with most current formulations would be difficult to deliver in a split dose.

*Practice point: starting dose in a patient on adequate LT4 monotherapy will always require removal of part of the LT4 dose and replacement with LT3. In practice the dose of LT3 will usually be a dose of 5 – 20 mcg a day in a split dose, by necessity often determined by the availability of low dose formulations of LT3.*

## Length of trial

**Fig. 2:**
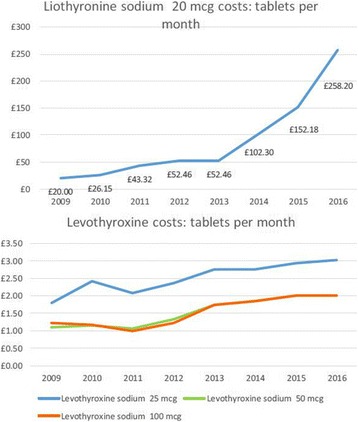
Proposed timeline for a trial of combination T4/T3 therapy

Again, there is very little evidence to determine how long a trial of combination therapy should be, the RCTs ranged from 5 weeks to 52 weeks. Most of those who showed a benefit had shown this by 3 months, and it is generally advised that 6 weeks to 3 months are waited until LT4 dose is adjusted to allow a steady state to develop in all tissues. It would therefore be reasonable to give a 3–6 month trial before deciding if it has been beneficial to the patient symptoms. If there is clear benefit it would be reasonable to continue the trial further, however, given the significant placebo effect seen in trials of thyroid hormone replacement [26] and the fact that LT3 may initially give a feeling of euphoria, clinicians should be encouraged to continue to assess the treatment as the benefit may disappear. The fixed term nature of the trial should be agreed with the patient prior to beginning and there should be agreement that there will be a return to LT4 monotherapy if no significant benefit is seen, given its easier dosing and better evidence of its safety. Figure 2 displays a proposed timeline for a trial.

*Practice point: initial trial 6 months, and then confirm benefit is still present at least 1 year before planning long-term therapy*

## Monitoring

There are no long term studies which link serum levels of T3 to adverse outcomes and therefore are able to direct monitoring of combination T4/T3 therapy. Given evidence for poorer outcomes with raised thyroid hormone levels/suppressed TSH levels in both subjects not on thyroid hormone replacement and on LT4 monotherapy it is reasonable to measure TSH, free T4 and free T3 2–4 h post dose as this is the expected peak of serum T3 post dose. Keeping both the serum T3 and TSH within the reference range at this point would suggest that the patient is at the least risk of developing both short and long term complications. Monitoring of these would include clinical assessment of pulse rate and rhythm, blood pressure, mood (particularly anxiety) and as clinically appropriate (depending on individual patient characteristics): ECG, echocardiogram and Bone Densitometry. Whilst studies in patients on LT4 would suggest that dose changes can be seen in end organ markers such as serum cholesterol and sex hormone binding globulin (SHBG), these changes are so small that they are often contained in the normal variance in the population.

*Practice point: Patients should be monitored indefinitely for cardiovascular, psychological and bone adverse effects.*

## Safety

Given the relatively small number of patients using combination T4/T3 therapy safety data has until recently been lacking. None of the 13 RCTs comparing combination therapy to T4 monotherapy showed any increase in adverse events in the combination group, however the follow up was generally short ranging from 5 to 52 weeks. Leese et al. have recently reported safety data in their observational cohort in the TEARS study [42]. In this study a subgroup of patients on thyroid hormone placement were considered with outcomes for those who had ever been taking LT3 (*n* = 400) compared to those who had only ever taken LT4 (*n* = 33,955). The study showed no increase in cardiovascular disease, atrial fibrillation or fractures (outcomes previously shown to be associated with levothyroxine overtreatment [78]) with a median follow-up of 9 years. There was an increased risk for new prescriptions of anti-psychotic medications of unclear significance. There was also a possible association with an increased risk of breast cancer in subjects who had taken LT3. Although there is some data on T3 augmentation of breast cancer cell line proliferation and higher endogenous T3 being associated with more aggressive breast cancers, the authors point out that this association was of borderline statistical significance and not related to number of prescriptions of LT3, arguing against a causal relationship. This potential association should be assessed in other large cohorts. The data from Leese et al. are from an observational study and so carry a risk of bias and self-selection; in addition the duration of LT3 use was not quantified. However, this may be the largest and longest duration study of its type for some time and offers some reassurance that the risks of taking T3 are not greater than expected.

## Cost

**Fig. 3:**
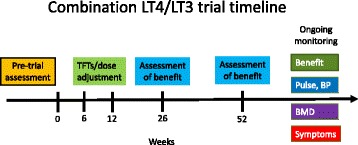
Change in costs for Liothyronine compared to Levothyroxine in the United Kingdom since 2009. Data from BNF and drug tariff, graph by British Thyroid Association

Lack of regulation of the cost of unbranded Liothyronine in the UK has seen the price rise markedly in recent years, currently 28 tablets of 20mcg costs around £258.20, therefore 10mcg bd for a year would cost around £3365 per patient. This represents a greater than 4000% increase from the branded ‘Tertroxin’ (just over £13 for 100 tablets) which precipitated a ‘black listing’ of the use of LT3 in several health trusts (Fig. 3) and has now attracted the attention of the UK regulatory and competition and markets authority. Synthetic T4 as Levothyroxine 100mcg once daily costs approximately £25 per patient per year. Whilst the government looks to close the loophole allowing companies to do this, many patients who had been maintained on LT3/LT4 for many years have been forced to come off LT3 or source it from overseas.

In Australia a similar dose of Liothyronine would cost $142 Australian dollars for a year on a private prescription, it is available on a government subsidized prescription for half that cost, available for patients who ‘have a documented intolerance or resistance to thyroxine’, which is open to the interpretation of the treating physician. A 100mcg tablet of thyroxine would cost around $58 Australia dollars a year on the government scheme.

In the US for patients not covered by health insurance, hypothyroid treatment typically costs $15–$100 per month -- or $180–$1200 per year -- for the synthetic thyroid hormone typically prescribed. For example, Drugstore.com charges about $15–$20, depending on the dose, for a one-month supply of the brand-name drug Levothroid, or $25–$45 for a one-month supply of the brand-name drug Synthroid. Drugstore.com charges up to $100 or more for a one-month supply, depending on dosage, of the brand-name drug Cytomel (LT3). Therefore in most countries combination therapy is comes at increased cost to the patient (or government) and this needs to be taken into consideration when planning a trial.

*Practice point: be aware and make patients aware of costs and availability of formulations prior to prescribing.*

## Dessicated thyroid extract

Many patients and alternative physicians prefer to use forms of dessicated thyroid extract (DTE) for thyroid hormone replacement. DTE is described as “the cleaned, dried, and powdered thyroid gland previously deprived of connective tissue and fat. It is obtained from domesticated animals that are used for food by humans” by the United States Pharmacopeia. Extracts of animal thyroids have been used for hypothyroid symptoms for many centuries in different cultures, and as a form similar to what is used today for over 110 years [79]. Most formulations are from porcine thyroid; however some from bovine thyroid or a combination of the two exist. Initially it was titrated to improvement in clinical symptoms and avoidance of hyperthyroid symptoms. The development of Levothyroxine and ability to assay serum TSH, T4 and T3 levels in the 1980’s led to calls for removal of DTE for treatment of hypothyroidism because of supraphysiological levels of T3 post dose, hyperthyroid symptoms and complications, and fluctuating levels of T3 as compared to very stable TSH, T3 and T4 levels with levothyroxine dosing [80–83]. Despite continuing concerns about these issues and also the consistency of the various preparations it continues to be used. Whilst the dramatic cases of thyroxtoxicosis on these preparations are mainly historical, there remain concerns about frequency of adverse events and calls for greater standardization of these preparations [84].

DTE is often prescribed in grains: 1 grain is typically around 60-65 mg of DTE and most commonly contains 38μg of T4 and 9μg of T3. Bioavailability has been shown to be different to synthetic LT4 and LT3 preparations [81]. Table 3 displays commonly available brands and doses contained. The doses above give a T4:T3 ratio of 4.2:1 significantly more T3 than the 14:1 secreted by the normal thyroid and the doses recommended above. This makes dosing difficult as displayed by several studies which have shown supraphysiological T3 doses post dose, fluctuating T3 levels during the day and more hyperthyroid symptoms in subjects taking DTE compared to LT4 monotherapy [80, 82, 85, 86]. As with combination T4/T3 therapy, there are no longer term trials or safety data available on long term use of DTE. This is important as there are concerns that regular supraphysiological levels of T3 may induce hyperthyroidism-like complications over the longer term. Given lack of safety data, difference and variability in the various preparations and no studies showing clear benefit over T4 monotherapy, all major endocrine and thyroid societies currently advise against the routine use of DTE for thyroid hormone replacement [5, 6].

**Table 3:** Dessicated thyroid extract formulations

Name	T3 dose	T4 dose	Available
Nature thyroid per 65 mg grain	9mcg	38mcg	US
Westhroid pure per 65 mg grain	9mcg	38mcg	US
NP thyroid per 60 mg grain	9mcg	38mcg	US
Thyroid (erfa) per 60 mg grain	8mcg	35mcg	Europe/Canada
Armour thyroid per 60 mg grain	9mcg	38mcg	US

Amour thyroid is the most commonly used formulation, also the most expensive, and in the US costs around $1 per 60 mg grain.. Price varies across the UK mostly around £1 per grain. These formulations are not available in Australia and therefore most users import them from the US at similar cost plus postage.

DTE formulations contain unmeasured quantities of diiodothyronine and monoiiodothyronine which many of its supporters believe make it a more suitable replacement for thyroid hormone, although there are no studies suggesting these are required for normal functioning, or that they are secreted in significant quantities from a normal human thyroid. These products also contain other thyroid-related proteins and antigens which could potentially invoke immune responses; these have not been studied to date.

*Practice point: whilst all major endocrine and thyroid societies advise against the use of DTE for hypothyroidism, it is clear that its use remains significant. Better regulated formulations and trials are required to determine its role, if any. Until these are available patients who continue to use DTE should be advised on appropriate safety monitoring as for using T4/T3.*

## Conclusion

Although there is convincing evidence that there is no benefit of combination T4/T3 therapy over T4 monotherapy for management of hypothyroidism at a population level, there remains a population of patients who do not feel well on T4 monotherapy. There are several possible reasons for this, one of which is an inability to use T4 effectively in a group of patients who may respond better to combination T4/T3 therapy. This will remain a possibility until large RCTs in an appropriately targeted population can confirm or refute it, and whilst it does a trial of combination therapy in such patients may be indicated. It is important that this be performed by a clinician with adequate knowledge and experience in the area, with appropriate patient selection, clear explanation of risks and benefits to the patient for consent and careful monitoring and follow-up. Possible ways to do this are covered in this review although it is clear that further research into this area and possible methods of delivering T3 are required.

## Abbreviations

ATA: American Thyroid Association
DTE: Dessicated Thyroid Extract
ECG: Electrocardiogram
ETA: European Thyroid Association
LT3: Liothyronine
LT4: Levothyroxine
mg: Milligram
RCT: Randomised Controlled Trial
SHBG: Sex Hormone Binding Globulin
T3: Tri-iodothyronine
T4: Thyroxine
TRH: Thyrotropin Releasing Hormone
TSH: Thyroid Stimulating Hormone
μg: Micrograms
UK: United Kingdom
US: United States (of America)
